# Anaphylactic reaction to intravenous diclofenac

**DOI:** 10.4103/0972-5229.78222

**Published:** 2011

**Authors:** Ranju Singh, Deepak Bansal, Neha Baduni, Homay Vajifdar

**Affiliations:** **From:** Department of Anesthesiology and Critical Care, Lady Hardinge Medical College & Associated Hospitals, New Delhi – 110 001, India

**Keywords:** Anaphylaxis, diclofenac sodium, intravenous, pulmonary embolism

## Abstract

Diclofenac sodium is a non-steroidal anti-inflammatory drug widely used as an opioid sparing agent for postoperative analgesia. Anaphylaxis due to intravenous diclofenac sodium is very rare. We report a case of anaphylactic reaction to IV diclofenac sodium, occurring postoperatively in a 25-year-old primigravida, the clinical features of which mimicked pulmonary embolism. The rarity, clinical importance and the diagnostic dilemma associated prompted us to report this case.

## Introduction

Diclofenac sodium is a non-steroidal anti-inflammatory drug (NSAID) widely used as an opioid sparing agent for postoperative analgesia. Commonly used routes of diclofenac administration are oral, intramuscular, intravenous, transdermal and rectal. Although anaphylaxis has been reported with oral,[1] intramuscular[2] and rectal routes[3] of administration of diclofenac, it has been rarely reported with intravenous administration. We report a case of severe hypersensitivity reaction to IV diclofenac, the clinical features of which mimicked pulmonary embolism. The rarity of the case prompted us to report this case.

## Case Report

A 25-year-old primigravida female was admitted with complaints of severe pain in the abdomen for 2 days and bleeding per vaginum for 1 day. When the patient arrived, she was drowsy, with a pulse rate (PR) of 150/minute, blood pressure (BP) of 84/56 mmHg, and SpO_2_ on air was 95%. She was diagnosed to have ruptured ectopic pregnancy and taken up for an emergency exploratory laparotomy. In view of excessive blood loss (3.0 l), she was shifted to Intensive Care Unit (ICU) postoperatively for elective ventilation. After 12 hours, the patient’s trachea was extubated. At that time, the vital parameters were PR 108/minute, BP 112/84 mmHg and SpO2 on air 100%. Four hours after extubation, IV diclofenac 75 mg was started in 100 ml of normal saline for postoperative analgesia. About 20-25 minutes after starting the infusion, the patient complained of tightness in her chest, palpitations and shortness of breath. Her saturation dipped to 55% on air, which increased to 72% on administering oxygen (FiO_2_ of 1.0), PR was 183/minute, BP was 80/40 mmHg, and respiratory rate (RR) was 40/minute. On examination, extremities were cold and auscultation of chest revealed bilateral ronchi. Her arterial blood gases demonstrated pH 7.47, pO_2_ 32 mm of Hg, pCO_2_ 37 mm Hg (Type I respiratory failure).

In view of these findings, pulmonary embolus or anaphylaxis to IV diclofenac was suspected. Infusion of diclofenac was stopped. Adrenaline (0.5 ml of 1:10,000) was injected intravenously. Patient’s trachea was intubated and she was put on a ventilator. Fluids were infused rapidly and vasopressor support (inj. dopamine 10 μg/kg/minute) was started to maintain hemodynamic stability. Low molecular weight heparin (enoxaparin sodium 40 mg subcutaneously), inj. methylprednisolone (250 mg IV), pheniramine (25 mg IV) and deriphylline (200 mg IV) were given and patient nebulized with respirator solution of salbutamol. Her saturation improved to 92% on FiO_2_ of 1.0, BP increased to 113/64 mmHg, PR decreased to 85/minute within about an hour of the onset of symptoms. Chest radiograph was done which showed bilateral pneumonitis. Bedside 2D echocardiography done at the same time showed a normal study. Laboratory investigations revealed elevated D-dimer (3.8 mg/l) and fibrinogen (5.1 g/l) levels. The prothrombin time (INR 1.3), hemogram, serum biochemistry and serum troponin T levels were normal. Serum tryptase levels done about an hour later were elevated (27 ng/ml). Methylhistamine level in the urinary sample taken about 4 hours later was 147 ng/μmol. Computed tomography (CT) pulmonary angiography that was done the next morning (about 12 hours after onset of symptoms) showed features suggestive of bilateral pneumonitis and no evidence of pulmonary embolism. CT venography of leg and pelvic veins was also done which did not reveal any evidence of deep vein thrombosis. The patient’s general condition and hemodynamic parameters improved gradually and her trachea was successfully extubated after 24 hours. She was discharged from the ICU to the ward the next day.

## Discussion

NSAIDs are amongst the most frequently used drugs that may cause hypersensitivity reaction. Anaphylaxis to diclofenac is an idiosyncratic reaction and is a rare event.[4] Safety data from clinical trials in the United States have shown that diclofenac sodium has lower rates of adverse reactions than any of the other comparative NSAIDs.[5]

Clinical features of anaphylaxis include increased vascular permeability, vasodilation, hypotension, tachycardia, bronchospasm, interstitial pneumonitis,[6] urticaria, angioedema, and even shock can occur. In the lung, bronchospasm, tissue edema and hypotension all contribute to impaired gas exchange resulting in hypoxia. Anaphylactic reactions have been reported to trigger cardiovascular events, including myocardial infarction and acute coronary syndromes, even in patients with normal coronary vasculature.[7] Pulmonary embolism can present with a wide range of clinical features many of which can also be seen in anaphylaxis, and differentiating between the two conditions can be challenging at times, more so in our patient who was a perfect candidate for developing pulmonary embolism (bedridden, dehydrated, hypercoagulable state). The other differential diagnoses considered were TRALI (hypoxemia, B/L chest infiltrates), and sudden acute reactionary hemorrhage (tachycardia, hypotension, but soft abdomen, no pallor) but were subsequently ruled out.

Both guidelines on pulmonary embolism and anaphylaxis emphasize the need for careful assessment of probability in making the diagnosis. Tryptase, a neutral protease selectively concentrated in the secretory granules of human mast cells (but not basophils), is released by mast cells together with histamine and serves as a marker of mast cell activation.[8] Another marker is urinary methylhistamine, a metabolite of histamine that has a longer half-life than histamine (2–3 hours).[9] With these markers, a retrospective diagnosis can be made after the initial treatment of the patient. Serum tryptase levels were elevated and methylhistamine was present in the urine in our patient, indicating anaphylactic reaction. Unfortunately, the patient did not come back for an epidermal skin prick testing which would have helped to confirm the diagnosis.

D-dimer testing is useful in excluding pulmonary embolism in patients, when used with assessment of clinical probability. However, it has poor specificity and is elevated in a variety of settings, including anaphylaxis, due to activation of the coagulation pathway by allergic mechanisms.[10] It is not surprising that high levels of D-dimer were found in the case reported here. The pulmonary infiltrates seen in our patient were probably caused by diclofenac administration as has been reported earlier.[11] CT (helical) pulmonary angiography, which is now the investigation of choice for the evaluation of suspected pulmonary embolism was done in our patient to rule out pulmonary embolism. Also, CT venography of leg and pelvic veins was done which did not reveal any evidence of deep vein thrombosis. Causal relationship between the diclofenac and event assessed using Naranjo probability scale[12] revealed a possible relationship (score +3).

Careful clinical assessment is important so that appropriate treatment can be instituted promptly to avoid further morbidity and mortality. Epinephrine is the first and most important treatment for anaphylaxis; considering its relative safety, when in doubt, epinephrine should be administered.[13] Also, an association between early anticoagulation and reduced mortality for patients with acute pulmonary embolism has been noted,[14] so anticoagulation can be started empirically when clinical features suggest acute pulmonary embolism in a patient.

To our knowledge, this is a rare case of anaphylactic reaction developing due to IV diclofenac. This communication is to bring awareness that although IV diclofenac sodium is a safe and widely used drug, severe and potentially fatal anaphylactic reactions may occur with its use. As it can present with features resembling pulmonary embolism, clinicians should be aware of this serious, albeit rare, complication of IV diclofenac to enable them to make a correct and timely diagnosis.
